# Secretory carrier-associated membrane protein 5 regulates cell-surface targeting of T-type calcium channels

**DOI:** 10.1080/19336950.2023.2230776

**Published:** 2023-06-30

**Authors:** Emilio R. Mustafá, Konstantin Weiß, Norbert Weiss

**Affiliations:** a Department of Pathophysiology, Third Faculty of Medicine, Charles University, Prague, Czech Republic; b Electrophysiology Laboratory of the Multidisciplinary Institute of Cell Biology (Argentine Research Council CONICET, Scientific Research Commission of the Buenos Aires Province and National University of La Plata, La Plata, Buenos Aires, Argentina

**Keywords:** Ion channels, Calcium channels, T-type channels, Secretory carrier-associated membrane protein 5, SCAMP5, Channelopathy

## Abstract

Missense mutations in the human secretary carrier-associated membrane protein 5 (SCAMP5) cause a variety of neurological disorders including neurodevelopmental delay, epilepsy, and Parkinson’s disease. We recently documented the importance of SCAMP2 in the regulation of T-type calcium channel expression in the plasma membrane. Here, we show that similar to SCAMP2, the co-expression of SCAMP5 in tsA-201 cells expressing recombinant Ca_v_3.1, Ca_v_3.2, and Ca_v_3.3 channels nearly abolished whole-cell T-type currents. Recording of intramembrane charge movements revealed that SCAMP5-induced inhibition of T-type currents is primarily caused by the reduced expression of functional channels in the plasma membrane. Moreover, we show that SCAMP5-mediated downregulation of Ca_v_3.2 channels is essentially preserved with disease-causing SCAMP5 R91W and G180W mutations. Hence, this study extends our previous findings with SCAMP2 and indicates that SCAMP5 also contributes to repressing the expression of T-type channels in the plasma membrane.

## Introduction

Low-voltage-activated T-type calcium channels play an essential role in shaping neuronal excitability [1]. While the contribution of T-type channels in the regulation of neuronal activity is intrinsically linked to their unique-gating properties, it also largely depends on the density of the channels in the plasma membrane and a number of signaling mechanisms ensure the proper expression of T-type channels at the cell surface [2]. For instance, several proteins including Kelch-like 1 [3,4], Stac1 [5], and USP5 [6] have been shown to interact with and potentiate the expression of T-type channels in the plasma membrane, while calnexin [7] and Rack-1 [8] in contrast inhibit their expression. Recently, we reported that the secretory carrier-associated membrane protein 2 (SCAMP2) is also associated with and regulates cell surface expression of T-type channels [9].

**Figure 1.**
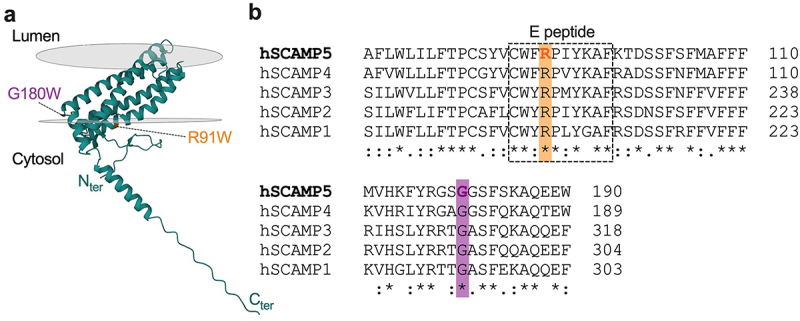
AlphaFold model of the human SCAMP5 showing the location of the R91W and G180W disease-causing mutations. (a) SCAMP5 is an integral membrane protein found essentially in secretory and endocytic organelles. It consists of a membrane core containing four transmembrane spans with cytoplasmic amino- and carboxy-termini, and a so-called E peptide essential to mediate SCAMP5 function. In this model, the R91W mutation (orange) is located on the cytosolic face of SCAMP5 while the G180W mutation (purple) is located in the intramembrane space of the protein. (b) Amino acid sequence alignment of human SCAMPs showing the conservation of the R91 and G180 residues across all SCAMP isoforms. Alignments were performed using UniProt (SCAMP1, O15126; SCAMP2, O15127; SCAMP3, O14828; SCAMP4, Q969E2; SCAMP5, Q8TAC9).

SCAMP proteins encompass a family of tetraspanning integral membrane proteins enriched in the trans-Golgi network and recycling endosomes and primarily implicated in vesicular trafficking and vesicle recycling processes [10]. SCAMPs consist of a membrane core containing four transmembrane spans with cytoplasmic amino- and carboxy-termini, and a so-called E peptide essential to mediate SCAMP function. Of the five known SCAMPs, SCAMP5 (Figure 1a-b) is highly expressed in the brain and plays an important role in the control of synaptic activity by regulating the sorting and trafficking of synaptic proteins [11–13]. Moreover, disease-causing SCAMP5 mutations were reported in patients with intellectual disability, seizure, and Parkinson’s disease [14–16], a clinical spectrum that overlaps with T-type calcium channelopathies [17].

In the present study, we hypothesized that similar to SCAMP2, SCAMP5 May contribute to the regulation of T-type channels, and that disease-causing SCAMP5 mutations could disrupt this regulation and contribute to SCAMP5-associated neurological manifestations. We show that SCAMP5 mediates downregulation of all three T-type channel isoforms (Ca_v_3.1, Ca_v_3.2, and Ca_v_3.3) by preventing the expression of the channels in the plasma membrane. However, this regulation is essentially preserved with disease-causing SCAMP5 mutations suggesting that T-type channels are likely to not contribute to SCAMP5-mediated neurological disorders.

## Material and methods

### Plasmid cDNA constructs and site-directed mutagenesis

The human wild-type SCAMP5 in pCMV3 was purchased from SinoBiological and was used as a template to generate the R91W and G180W mutants by site-directed mutagenesis (performed by GenScript). The fidelity of all constructs was confirmed by full-length sequencing of the coding region. The human Ca_v_3.1, Ca_v_3.2, and Ca_v_3.3 in pcDNA3.1 were previously described [18].

### Cell culture and heterologous expression

Human embryonic kidney tsA-201 cells were grown in DMEM medium supplemented with 10% fetal bovine serum and 1% penicillin/streptomycin (all media purchased from Invitrogen) and maintained under standard conditions at 37°C in a humidified atmosphere containing 5% CO_2_. Heterologous expression was performed by transfecting cells with plasmid cDNAs encoding for Ca_v_3.1/Ca_v_3.2/Ca_v_3.3 and SCAMP5 variants in a 1:1 ratio and empty pEGFP vector as transfection marker using the calcium/phosphate method.

### Patch clamp electrophysiology

Patch clamp recordings of T-type currents in tsA-201 cells were performed 72 h after transfection in the whole-cell configuration at room temperature (22–24°C) in a bath solution containing (in millimolar): 5 BaCl_2_, 5 KCl, 1 MgCl_2_, 128 NaCl, 10 TEA-Cl, 10 D-glucose, 10 4-(2-hydroxyethyl)-1-piperazineethanesulfonic acid (HEPES) (pH 7.2 with NaOH). Patch pipettes were filled with a solution containing (in millimolar): 110 CsCl, 3 Mg-ATP, 0.5 Na-GTP, 2.5 MgCl_2_, 5 D-glucose, 10 EGTA, and 10 HEPES (pH 7.4 with CsOH), and had a resistance of 2–4 MΩ. The linear leak component of the current was corrected using a P/4 subtraction protocol and current traces were digitized at 10 kHz and filtered at 2 kHz. The voltage dependence of activation was determined by measuring the peak of the T-type current in response to depolarizing steps from −80 mV to +20 mV applied every 5 sec from a holding membrane potential of −100 mV. The current-voltage relationship (*I*/*V*) curve was fitted with the following modified Boltzmann Equation (1):

(1) $$I\left(V\right)=Gmax\frac{\left(V-Vrev\right)}{1+\;exp\frac{\left(V0.5-V\right)\;}{k}}$$

with *I*(*V*) being the peak current amplitude at the command potential *V*, *G*_max_ the maximum conductance, *V*_*rev*_ the reversal potential, *V*_0.5_ the half-activation potential, and *k* the slope factor. The voltage dependence of the whole cell Ba^2+^ conductance was calculated using the following modified Boltzmann Equation (2):

(2) $$G\left(V\right)=\;\frac{Gmax}{1+\;exp\frac{\left(V0.5-V\right)\;}{k}}$$

with *G*(*V*) being the Ba^2+^ conductance at the command potential *V*.

Recording of intramembrane charge movement was performed in an external solution containing (in mM): 95 CsCl, 40 TEACl, 5 BaCl_2_, 1 MgCl_2_, 10 D-glucose, 10 HEPES (pH 7.4 with CsOH). Patch pipettes were filled with a solution containing (in mM): 130 CH_3_SO_3_Cs, 5 Na-ATP, 5 MgCl_2_, 10 TEA-Cl, 10 EGTA, 10 HEPES (pH 7.4 with CsOH). Only cells with an input resistance less than 5 MΩ were considered. The remaining artifacts were subtracted using a P/4 procedure. ON-gating currents (Q_on_) were recorded in response to a series of 5 depolarizing pulses at the reversal potential of the ionic current assessed for each cell, and total gating charge Q_rev_ was calculated as the integral of the area below the averaged current traces. The time course of Q_on_ integral was used to determine 10–90% rise time of intramembrane charge movement.

All recordings were performed using an Axopatch 200B amplifier (Axon Instruments) and acquisition and analysis were performed using pClamp 10 and Clampfit 10 softwares, respectively (Axon Instruments).

### Statistical analysis

Data values are presented as violin plots. Statistical analysis was performed using GraphPad Prism 7. For datasets passing the D’Agostino & Person omnibus normality test, statistical significance was determined using a Mann–Whitney test. For multiple comparison analyses, Kruskall-Wallis with Dunn’s posttest was used. Datasets were considered to be significantly different for *p* < 0.05 *.

## Results

### SCAMP5 reduces T-type current density

**Figure 2.**
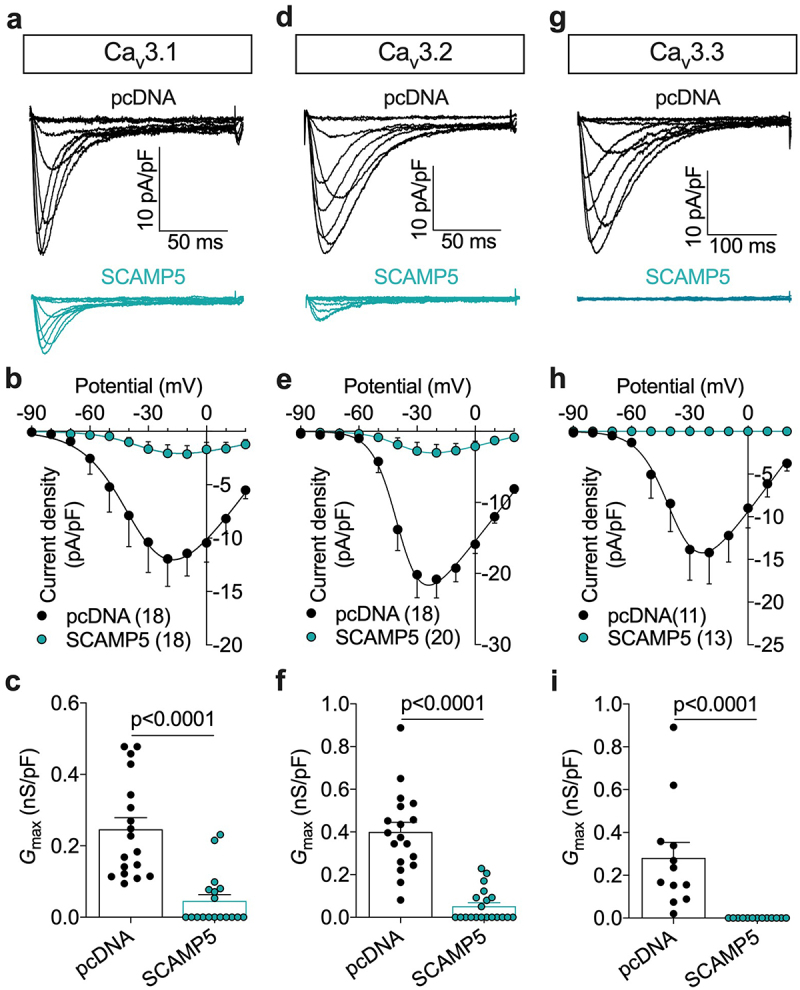
SCAMP5 reduces T-type channel current density. (a) Representative whole-cell T-type current traces recorded in Ca_v_.1-expressing tsA-201 cells cotransfected with either empty pcDNA plasmid (black traces) or with wild-type SCAMP5 (green traces). Currents were elicited by depolarizing steps to values ranging between −90 mV and +20 mV from a holding potential of −100 mV. (b) Corresponding mean current/voltage (I/V) relationships. The continuous lines represent the fit of the I/V curves with the modified Boltzmann Eq. (1). (c) Corresponding mean maximal macroscopic conductance values (G_max_). (d-f) and (g-i) Legend same as (a-c) but for cells expressing Ca_v_.2 and Ca_v_.3 channels, respectively. Numbers in parentheses indicate the number of cells recorded per condition. Data are presented as mean ± SEM for (n) recorded cells.

To assess the role of SCAMP5 in the regulation of T-type channels, we performed patch clamp recordings of T-type currents in tsA-201 cells expressing recombinant Ca_v_3.1, Ca_v_3.2, and Ca_v_3.3 channels. We observed that co-expression of wild-type (WT) SCAMP5 caused an almost complete drop of the whole-cell T-type current in cells expressing Ca_v_3.1 (Figure 2a-b) and Ca_v_3.2 channels (Figure 2d-e). For instance, the maximal macroscopic conductance (*G*_max_) was reduced by 80% (from 0.25 ± 0.03 nS/pF, *n* = 18 to 0.05 ± 0.02 nS/pF, *n* = 18; *p* < 0.0001) in cells expressing Ca_v_3.1 (Figure 2c and Table 1) and by 87% (from 0.40 ± 0.04 nS/pF, *n* = 18 to 0.05 ± 0.02 nS/pF, *n* = 20; *p* < 0.0001) in cells expressing Ca_v_3.2 (Figure 2f and Table 1) without alteration of the voltage dependence of activation of the channels (Table 1). In cells expressing Ca_v_3.3 channels, the co-expression of SCAMP5 entirely abolished the T-type conductance (Figure 2g-i).

**Table 1.** Steady-state activation properties of recombinant T-type channels expressed in tsA-201 cells in the presence of SCAMP5. Data are presented as mean ± SEM for (n) recorded cells. G_max_, maximal macroscopic conductance; V_0.5_, half activation potential; k, activation slope factor; ND; not determined.

	*G*_max_ (nS/pF)	*p*	*V*_0.5_ (mV)	*p*	*k* (mV)	*p*	(n)
Ca_v_3.1	0.25 ± 0.03		−30.22 ± 4.33		7.49 ± 0.64		18
SCAMP5	0.05 ± 0.02	<0.0001*	−28.43 ± 5.75	0.8224	10.06 ± 1.30	0.0589	18
Ca_v_3.2	0.40 ± 0.04		−33.82 ± 2.60		5.56 ± 0.74		18
SCAMP5	0.05 ± 0.02	<0.0001*	−30.56 ± 3.07	0.4954	8.84 ± 1.05	0.0133	20
Ca_v_3.2	1.04 ± 0.13		−28.52 ± 1.34		10.13 ± 0.57		15
R91W	0.08 ± 0.04	<0.0001*	−41.51 ± 4.29	0.0253*	10.12 ± 1.34	>0.9999	13
G180W	0.00 ± 0.00	<0.0001*	ND	ND	ND	ND	12
Ca_v_3.3	0.28 ± 0.07		−36.68 ± 2.55		6.46 ± 0.84		11
SCAMP5	0.00 ± 0.00	<0.0001*	ND	ND	ND	ND	13

### SCAMP5 represses cell surface expression of T-type channels

**Figure 3.**
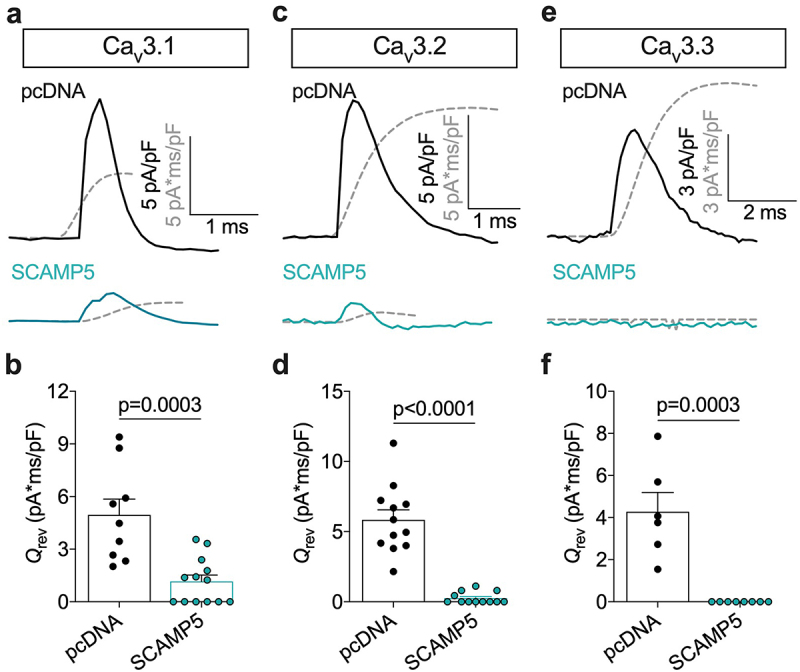
SCAMP5 reduces intramembrane charge movement. (a) Representative intramembrane on charge movement traces recorded at the ionic reversal potential in Ca_v_.1-expressing tsA-201 cells cotransfected with either empty pcDNA plasmid (black traces) or with wild-type SCAMP5 (green traces). The dotted lines depict the time course of the intramembrane charge movement integral. (b) Corresponding mean intramembrane charge movement values (Q_rev_). (c-d) and (e-f) Legend same as (a-b) but for cells expressing Ca_v_.2 and Ca_v_.3 channels, respectively. Numbers in parentheses indicate the number of cells recorded per condition. Data are presented as mean ± SEM for (n) recorded cells.

The reduced T-type conductance in the presence of SCAMP5 is either caused by an alteration of the channel gating or by a reduced expression of the channel proteins at the cell surface. Therefore, we next analyzed intramembrane charge movements to assess the density of channels in the plasma membrane. Intramembrane charge movements were assessed at the ionic reversal potential (*Q*_rev_) which provides an estimate of the total number of functional channels embedded in the plasma membrane. Consistent with the reduced T-type conductance, the co-expression of SCAMP5 produced a strong reduction of *Q*_rev_ in cells expressing Ca_v_3.1 (Figure 3a) and Ca_v_3.2 channels (Figure 3c). For instance, *Q*_rev_ was reduced by 67% (from 4.96 ± 0.90 pA*ms/pF, *n* = 9 to 1.62 ± 0.36 pA*ms/pF, *n* = 13; *p* < 0.0003) in cells expressing Ca_v_3.1 (Figure 3b and Table 2) and by 95% (from 5.85 ± 0.71 pA*ms/pF, *n* = 12 to 0.26 ± 0.12 pA*ms/pF, *n* = 12; *p* < 0.0001) in cells expressing Ca_v_3.2 (Figure 3d and Table 2). In cells expressing Ca_v_3.3, and consistent with the absence of ionic T-type conductance, we did not detect any charge movement (Figure 3e-f and Table 2).

**Table 2.** Electrophysiological properties of intramembrane charge movements recorded in tsA-201 cells expressing recombinant T-type channels in the presence of SCAMP5. Data are presented as mean ± SEM for (n) recorded cells. Q_rev_, gating charge at the reversal ionic potential; ND; not determined.

	*Q*_rev_ (pA*ms/pF)	*p*	10–90% rise (ms)	*p*	± decay (ms)	*p*	(n)
Ca_V_3.1	4.96 ± 0.90		0.52 ± 0.06		0.39 ± 0.05		9
SCAMP5	1.62 ± 0.36	0.0003*	1.28 ± 0.38	0.0389*	0.64 ± 0.08	0.0150*	13
Ca_V_3.2	5.85 ± 0.71		1.03 ± 0.13		0.57 ± 0.07		12
SCAMP5	0.26 ± 0.12	<0.0001*	0.70 ± 0.19	0.0589	0.59 ± 0.17	0.8775	12
Ca_V_3.3	4.28 ± 0.91		2.07 ± 0.63		1.37 ± 0.33		6
SCAMP5	0.00 ± 0.00	<0.0001*	ND	ND	ND	ND	8

### SCAMP5 R91W and G180W variants retain their ability to modulate Ca_v_.2 channels

**Figure 4.**
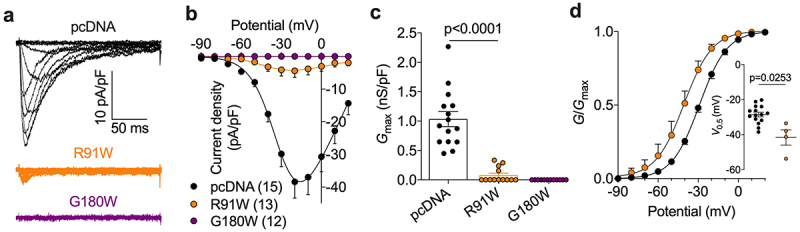
SCAMP5 R91W and G180W variants retain their ability to downregulate Ca_v_.2 channels. (a) Representative whole-cell T-type current traces recorded in Ca_v_.2-expressing tsA-201 cells cotransfected with either empty pcDNA plasmid (black traces) or in combination with SCAMP5 R91W (orange traces) and G180W (purple traces) variants. (b) Corresponding mean current/voltage (I/V) relationships. The continuous lines represent the fit of the I/V curves with the modified Boltzmann Eq. (1). (c) Corresponding mean maximal macroscopic conductance values (G_max_). (d) Corresponding mean normalized voltage dependence of activation for Ca_v_.2 alone (black symbols) and in combination with SCAMP5 R91W variant (orange symbols). The continuous lines represent the fit of the conductance curves with the modified Boltzmann Eq. (2). The inset shows the corresponding mean half-activation potential (V_0.5_ activation) values. Data are presented as mean ± SEM for (n) recorded cells.

In another set of experiments, we aimed to assess whether SCAMP5-dependent regulation of T-type channels is affected by disease-causing SCAMP5 R91W and G180W mutations (Figure 1a). The R91W mutation was reported to cause pediatric epilepsy [14] and is located in the so-called E peptide (Figure 1a-b), a highly conserved region across SCAMP isoforms and essential for their functional activity [19]. In contrast, the G180W mutation was associated with neurodevelopmental disorder with autistic features and seizures [16] and is located in a variable region of the protein (Figure 1a-b). Given that T-type currents recorded in the presence of SCAMP5 were small, the concentration in divalent was brought to 10 mM in order to increase the magnitude of the T-type conductance and allow for a better analysis. Similar to what we observed with SCAMP5 WT, co-expression of SCAMP5 R91W in cells expressing Ca_v_3.2 produced a 92% reduction of the T-type conductance (from 1.04 ± 0.13 nS/pF, *n* = 15 to 0.08 ± 0.04 nS/pF, *n* = 13; *p* < 0.0001) while in cells co-expressing SCAMP5 G180W variant the T-type current remained below the detection threshold (Figure 4a-c and Table 1). Moreover, and in contrast to SCAMP5 WT, the R91W mutation produced an additional 13 mV hyperpolarizing shift of the voltage dependence of activation of Ca_v_3.2 (from −28.52 ± 1.34 nS/pF, *n* = 15 to −41.51 ± 4.29 nS/pF, *n* = 4; *p* < 0.0253) (Figure 4d and Table 1). However, while this additional gating effect may indeed reflect an alteration in SCAMP5-dependent regulation of Ca_v_3.2 channels, it is likely to not have a physiological consequence given the calcium conductance is already nearly abolished.

## Discussion

Previous studies have documented the role of SCAMP proteins in the sorting and surface targeting of ion channels and receptors. For instance, SCAMP2 was reported to regulate cell surface expression and localization of the sodium-potassium-chloride cotransporter 2 (NKCC2) [20], as well as the dopamine (DAT) [21] and serotonine (SERT) [22] transporters. In addition, SCAMP2 also regulates cell surface targeting of the sodium-proton (Na^+^/H^+^) exchanger [23] and recently, we have reported its role in the expression of T-type calcium channels [9]. In this study, we showed that reminiscent to SCAMP2, SCAMP5 acts as a potent repressor of the expression of T-type channels in the plasma membrane irrespective of the T-type channel isoform. The observation that both the T-type conductance and intramembrane charge movements were concomitantly reduced upon co-expression of SCAMP5 indicates that this regulation mostly occurs by repression of the expression of the channels in the plasma membrane. While we have not directly measured the expression level of T-type channels when co-expressed with SCAMP5, it is likely to not be at the origin of the reduced T-type conductance since this effect is reminiscent to what we previously observed with SCAMP2 where the co-expression did not affect the level of channel proteins [9].

Given that SCAMP5 and Ca_v_3.2 channels are both candidate genes for epilepsy [17], and SCAMP5 R91W knock-in mice showed increased neuronal excitability [14], we also assessed the regulation of Ca_v_3.2 in the presence of disease-causing SCAMP5 mutations. We showed that SCAMP5-mediated downregulation of Ca_v_3.2 channels is essentially preserved with disease-causing SCAMP5 R91W and G180W mutations. While the E peptide was previously reported to support SCAMP2-mediated downregulation of several ion channels and transporters including T-type channels, this regulation is largely mediated by the proximal cysteine (C) and tryptophane (W) residues [9,20,22] (see Figure 1b). This likely explains why the R91W mutation that affects a downstream residue within the E peptide did not alter SCAMP5-dependent regulation of Ca_v_3.2 channels. However, and in contrast to SCAMP5 WT, the R91W mutation produced an additional hyperpolarizing shift of the voltage dependence of the activation of Cav3.2. While SCAMP proteins are essentially expressed in the Golgi network, a previous study reported the existence of a small population of SCAMP1 and 2 in the plasma membrane [19]. It is therefore a possibility that SCAMP5 R91W may also translocate to the plasma membrane where it may mediate its gating effect on the channel. Nonetheless, this additional gating effect on the channel is not anticipated to have any physiological consequence given the primary effect of SCAMP5 to nearly abolish the T-type conductance.

In summary, this study identifies SCAMP5 as a potent repressor of the expression of T-type channels in the plasma membrane and add to the notion that SCAMP proteins are important regulators of the expression of T-type channels. While this regulation appears to be preserved by disease-causing SCAMP5 R91W and G180W mutations, additional analysis on native T-type channels would nonetheless be necessary and may potentially uncover more subtle effects of these mutations. For instance, SCAMP5 was reported to engage with the synaptic vesicle release machinery by interacting with SNARE proteins [24]. Considering that T-type channels form a functional signaling complex with syntaxin 1A [25], it is a possibility that SCAMP5 May also exert additional effects on the channels by virtue of SNARE proteins.

## Data Availability

All data generated or analyzed during this study are included in this published article and its supplementary information files.

## List of abbreviations

Ca_v_: Voltage-gated calcium channel
DAT: Dopamine transporter
*G*_max_: Maximal macroscopic conductance
NKCC2: Sodium-potassium-chloride cotransporter 2
*Q*_rev_: Intramembrane charge movement at the reversal ionic potential
Rack-1: Receptor for activated C kinase 1
SCAMP2: Secretory carrier-associated membrane protein 2
SCAMP5: Secretory carrier-associated membrane protein 5
SERT: Serotonine transporter
SNARE: Soluble N-ethylmaleimide sensitive factor attachement protein receptor
USP5: Ubiquitin specific peptidase 5
